# Coadsorption behaviors and mechanisms of Pb(ii) and methylene blue onto a biodegradable multi-functional adsorbent with temperature-tunable selectivity

**DOI:** 10.1039/d0ra07139k

**Published:** 2020-09-28

**Authors:** Rongping Chen, Jiali Cai, Qing Li, XinYuan Wei, Huihua Min, Qiang Yong

**Affiliations:** College of Biology and Environment, Nanjing Forestry University Nanjing 210037 P. R. China; The Key Laboratory of Chemistry for Natural Products of Guizhou Province, Chinese Academy of Sciences Guiyang 550014 China; Electron Microscope Lab, Nanjing Forestry University Nanjing 210037 P. R. China; College of Chemical Engineering, Nanjing Forestry University Nanjing 210037 P. R. China swhx@njfu.com.cn +86-25-85427045

## Abstract

An entirely bio-degradable adsorbent based on lignin was synthesized by a crosslinking method and the adsorption of methyl blue (MB) and Pb(ii) onto the adsorbent were comparatively investigated, with adsorption behavior and mechanism of the two pollutants on the adsorbent (SLS) being assessed in single and binary systems. According to the results, SLS was capable of effective adsorption using MB and Pb(ii). The adsorption behavior of MB and Pb(ii) followed Langmuir and pseudo-first order models and showed temperature-dependent preferences. At 298 K MB was more preferred while at 318 K Pb(ii) adsorption was more favorable, which means that the selectivity of SLS can be tuned by changing the temperature. From a mechanism aspect, the adsorption of MB and Pb(ii) were both achieved through more than one route. Pb(ii) mainly interacts with sulfonate and hydroxyl groups on SLS, while MB can be bound on both anionic and aromatic groups due to its aromatic nature. Recycling and reuse experiments showed that used SLS can be readily reactivated and stably reused. The findings will guide adsorbent applications in wastewater containing heavy metals and aromatic compounds.

## Abstract 2

The preparation mechanism of SLS.

## Graphical abstract

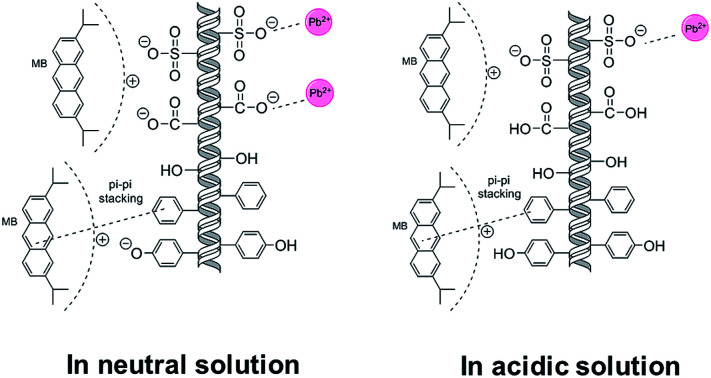

## 1. Introduction

Heavy metal and dye compound pollution have always been paid considerable attention.^1–6^ Heavy metals and dyes usually coexist in industrial wastewaters, resulting in their combined toxic effects on human beings and ecosystems. Therefore, it is of great importance to separate and treat them in wastewater. Traditionally, various methods, including adsorption, flocculation, oxidation and biotechnology, are adopted to remove pollutants from wastewater.^7–11^ Each technique has its features and suitable applications. Among these methods, it is more attractive to perform the coadsorption of heavy metals and dyes with a kind of versatile adsorbent.^4,12–15^

In recent years, concern was focused on the secondary pollution of adsorption, and more attention has been paid in completely green treatment of water using natural polymers, which is eco-friendly to environment.^16^ Starch is one of the most abundant biomaterials on the earth,^17^ its biodegradability and modification convenience^18^ make it stand out among polysaccharides such as cellulose.^17^ Lignosulfonate is another bio-based material that is gaining wide use as flotation agent and dispersant.^19,20^ It bears good chelating and ion-exchange affinity with multiple metal ions and dyes,^12^ but its solubility in water makes it difficult to directly apply as an adsorbent. Only with proper immobilization it can serve as a fine adsorbent. Starch, as a fully biodegradable material that is also readily modifiable, is ideal to immobilize lignosulfonate.

In this study, a novel versatile adsorbent based on starch and lignosulfonate was prepared using crosslinking technique. Both raw materials are inexpensive and biodegradable natural products. The characteristics of the two materials are complementary to each other: starch is easy to access but inactive in adsorption, while lignosulfonate shows ion exchange and chelating activities but dissolves in water. In addition, the methods of synthesis are more eco-friendly, compared with other conventional modifications. The adsorption behavior of the adsorbent to two typical pollutants, namely methylene blue and Pb(ii), was systematically assessed to evaluate its application potential. The two pollutants are selected as typical models for dyes and heavy metals, as they may coexist in wastewater, leading to extensive environmental hazard and health concern.^21^ The adsorption behavior of the adsorbent (SLS) for removal of two kinds of pollutants has been performed to assess its versatility. Meanwhile, the selective adsorption mechanism in binary systems has been investigated.

## 2. Material and methods

### 2.1 Materials

The materials for SLS preparation, namely sodium lignosulfonate (LS, 3.55% S content), epichlorohydrin, and starch was supplied by Sinopharm Chemical Reagent Co., Ltd., China. Hydrochloric acid (HCl), sodium hydroxide (NaOH), methylene blue, Pb(NO_3_)_2_ and cyclohexane were purchased from Nanjing Chemical Reagent Co., Ltd., China. Deionized water was used in all experiments.

### 2.2 Preparation of adsorbents

**Scheme 1:**
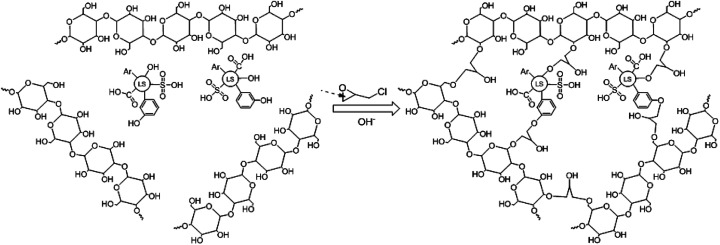
The preparation mechanism of SLS.

SLS was synthesized by the method of inverse cross-linking. In SLS preparation, firstly, sodium lignosulfonate (2.0 g) and starch (3.0 g) were dissolved in 50 mL of deionized water and NaOH (1.2 g) was subsequently added to activate the starch, the reactor was also heated to 353 K under stirring to enhance starch dissolution. Then, epichlorohydrin (3.0 g) was added to the water phase and the water phase was poured into 300 mL of cyclohexane (containing 1.0 g of Span-80 and additional 2.0 g of epichlorohydrin) solution under fast stirring. The suspension was kept for crosslinking for 24 h at room temperature. The crosslinked products, named SLS microspheres, were washed with ethanol to remove excess oil phase and surfactant. Finally, SLS was reserve in water for following use. The synthesis mechanism of SLS microspheres was illustrated in Scheme 1.

### 2.3 Characterization of SLS

SLS was characterized *via* multiple techniques: Fourier transform infrared spectroscopy (FTIR, on Avatar360; Nicolet Co.; USA); zeta potential analysis (on Nano ZS90; Marvin Co.; UK, pH 1–9); field emission scanning electron microscope (FSEM, on JSM-7600F; JEOL Co.; Japan); energy dispersive spectrometer (EDS, on INCA X-ACT; Oxford Co.; UK); elemental analysis (on PE-2400II; PE Co.; USA).

### 2.4 Adsorption of MB and Pb(ii) in sole component system

The adsorption experiments were conducted in sole component system. *q* (mg g^−1^), was calculated from eqn (1):1
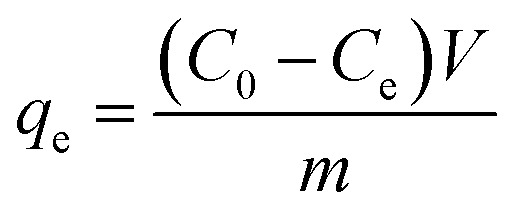
where *C*_0_ and *C*_e_ (mg L^−1^) are the concentration of the target pollutants at initial and equilibrium time, respectively; *V* (L) and *m* (g) refer to the volume of absorbate solution and the dried mass of the adsorbent, respectively.

The concentrations of MB and Pb(ii) in the supernatant were analyzed by Vis spectrometer (Victor 722) at 662 nm and atomic absorption spectrophotometer (PE AA900T), respectively.

To avoid the precipitation of adsorbate in alkaline solution, the initial pH were adjusted about 0.8–9 for MB and 0.8–6 for Pb(ii) by HNO_3_ or NaOH. 0.05 g of SLS was added to the glass flask and mixed in 50 mL solution with different initial pH values. And then these flasks were shaken at 160 rpm for 24 h at 298 K to attain equilibrium. Meanwhile, the adsorption experiments onto starch were performed in the same method.

In adsorption equilibrium studies, 0.05 g of SLS was added in 50 mL of Pb/MB solutions which their initial concentrations ranged from 10 to 400 mg L^−1^. The experiments were also performed at 298 K at pH 5.0, shaking for 24 h to attain adsorption equilibrium.

Kinetic adsorption experiments were also performed at 298 K with pH 5.0. Adsorbents were weighed and added to MB/Pb solutions with continuous stirring. Every preset time intervals, 1 mL of supernatant were pumped out to analyze the instant concentration of pollutant. And then equal volume of distilled water was instantly poured into the system to maintain volume constant. The pollutant uptake at time *t*_*i*_, *q*(*t*_*i*_) (mg g^−1^), was obtained according to eqn (2):2
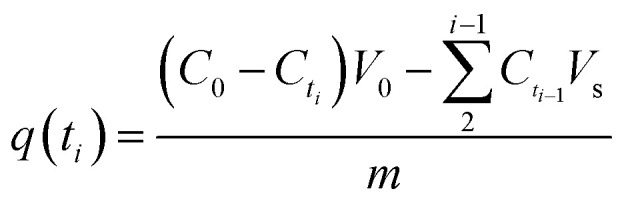
where *C*_*t*_*i*__ (mg L^−1^) is the concentrations at time *t*_*i*_ (min). *V*_0_ and *V*_s_ (L) refer the initial volume of the pollutant solution and the volume taken out each time for concentration analysis, respectively. *V*_s_ is set to 1 mL.

### 2.5 Adsorption in bi-component system

In order to evaluate the impact of SLS in wastewater to multiple pollutants, the adsorption of Pb/MB in a coexistent system was carried out, the experiments were conducted at 298 K.

### 2.6 Recycling experiments

Reusability of adsorbents is also vital for its application. The reusability of Pb and MB loaded on SLS was verified by recycling experiments. The recycling experiments were performed at room temperature. The adsorbents loading adsorbates were regenerated with 0.1 M HNO_3_ followed by re-activation by 0.1 M NaOH. Then the recovered adsorbents were filtrated and washed with ultrapure water. The regenerated adsorbents were reused in the next cycle of adsorption experiments. In an adsorption–desorption cycle, the adsorbent was saturated in pollutant solution and then filtered, recovered, and reused in the next cycle again. The recycling was repeated for five cycles. The recycle efficiency (*R*%) are calculated according to the following eqn (3):3
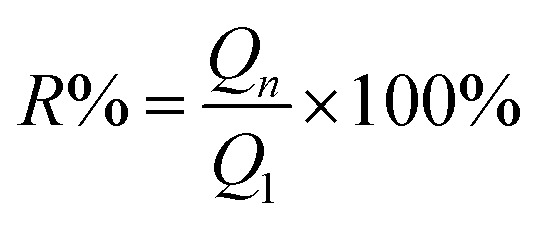
where *Q*_1_ and *Q*_*n*_ refer the adsorption capacity of SLS in the first and *n* cycle, respectively.

## 3. Results and discussions

### 3.1 Characterization of the adsorbent

**Fig. 1:**
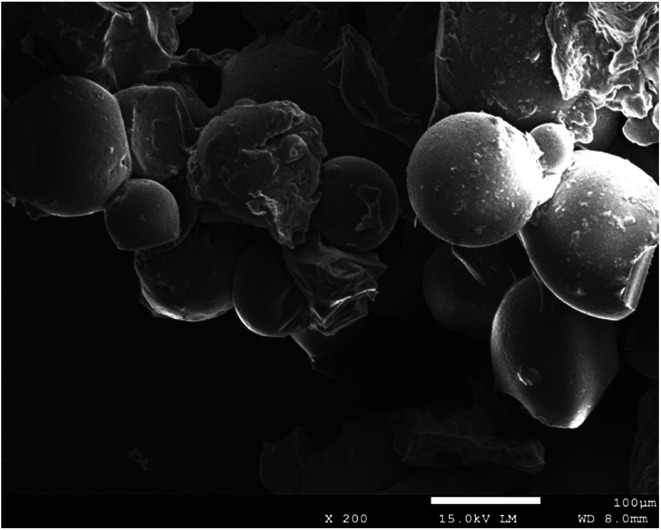
The SEM image of SLS.

The water content of SLS was about 82.3%. After being sputter coated with gold, the surface morphologies of these gel beads were observed directly by SEM, which was shown in Fig. 1. It was found that the appearance of SLS particles were quasi-spherical and primrose yellow color. According to Fig. 1, they are gel beads with sizes of about 100 μm.

**Fig. 2:**
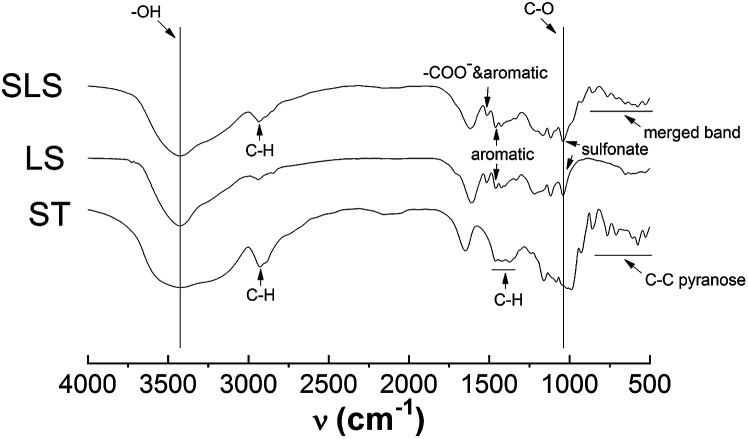
IR spectra of SLS.

Fig. 2 showed the IR spectra of SLS, as can be observed, characteristic bands from lignosulfonate and starch, such as hydroxyl band at 3200–3500 cm^−1^ (ST&LS), C–O band at 1000–1100 cm^−1^ (ST&LS), aromatic C=C band nearly at 1400 cm^−1^ and 1600 cm^−1^ (carboxyl & aromatic at 1600 cm^−1^). Compared with ST, SLS and LS observed a typical absorption peak of sulfonate (about 1060 cm^−1^ and 1160 cm^−1^) and aromatic groups (1400 cm^−1^ and 1600 cm^−1^). The band nearly at 1650 cm^−1^ (in ST) is probably the binded water in it. Besides, in SLS, there are adjacent bands of the different groups from LS and ST merging with each other, such as the carboxyl & aromatic and finger-print zone bands. From the spectra of SLS, typical peaks of sulfonate (1060 cm^−1^ and 1160 cm^−1^) and aromatic groups (1400 cm^−1^ and 1600 cm^−1^)^2^ appeared in the spectrum of SLS, indicating the successful immobilization of LS by ST.

The degree of LS grafting was further determined by sulfur content from elemental analysis, as the sulfur content of SLS suggest that about 30% mass in SLS is LS. Sulfonate content in SLS, according to Table 1, was about 0.35 mmol g^−1^.

**Table 1:** Elemental analysis results of SLS

Element content (%)	ST	LS	SLS
C	42.68	43.74	47.22
H	6.42	4.77	6.64
S	0	3.55	1.12
O	50.60	36.30	44.19
Sulfonate (mmol g^−1^)	0	1.11	0.35

**Fig. 3:**
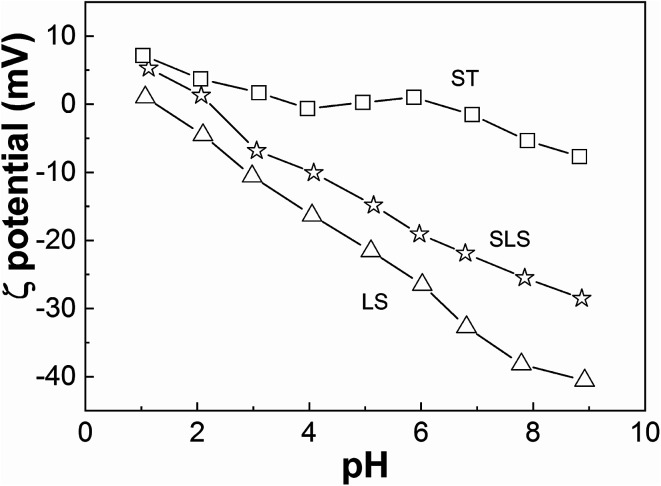
Zeta potential *versus* pH.

Zeta potential of SLS, LS and ST were compared in Fig. 3. The zeta potential of ST stays near zero point except in strongly acidic or alkaline conditions, while those of LS keeps negative value with the pH ranging from 1 to 9 owing to the sulfonate groups in LS. The isoelectric point of SLS was about at pH 2, confirming the successful integration between LS with starch.

**Fig. 4:**
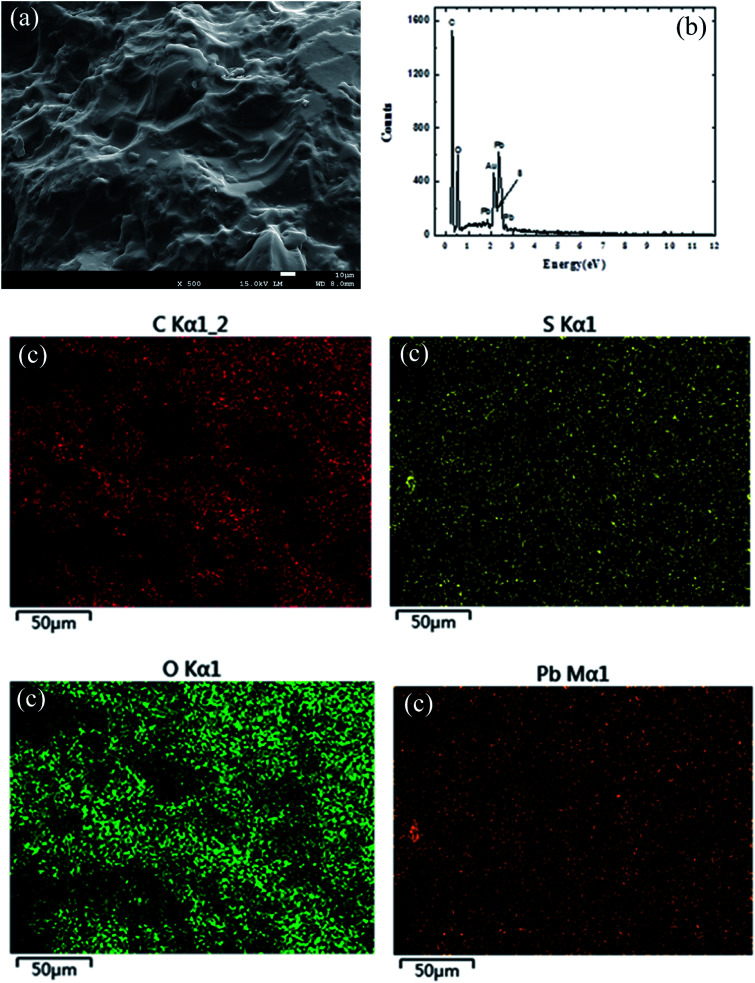
The SEM of Pb(ii) on SLS (a); the EDS of Pb(ii) on SLS (b); the mapping of Pb(ii) on SLS (c).

In order to further study the composition of SLS, the element analysis of SLS was carried out. These include morphology of Pb(ii) adsorption on SLS such as SEM (Fig. 4(a)), mapping test (Fig. 4(c)) and EDS test (Fig. 4(b)). The EDS figure showed that the samples mainly contain C, O, S and Pb (adsorption on SLS) elements. Fig. 4(b and c) confirmed the successful synthesis of starch and lignosulfonate, and its effective adsorption to Pb(ii).

As the above characterization data suggest, LS has been successfully immobilized onto ST using the aforementioned method.

### 3.2. Adsorption behaviors of the adsorbent

#### 3.2.1 pH effect on adsorption

**Fig. 5:**
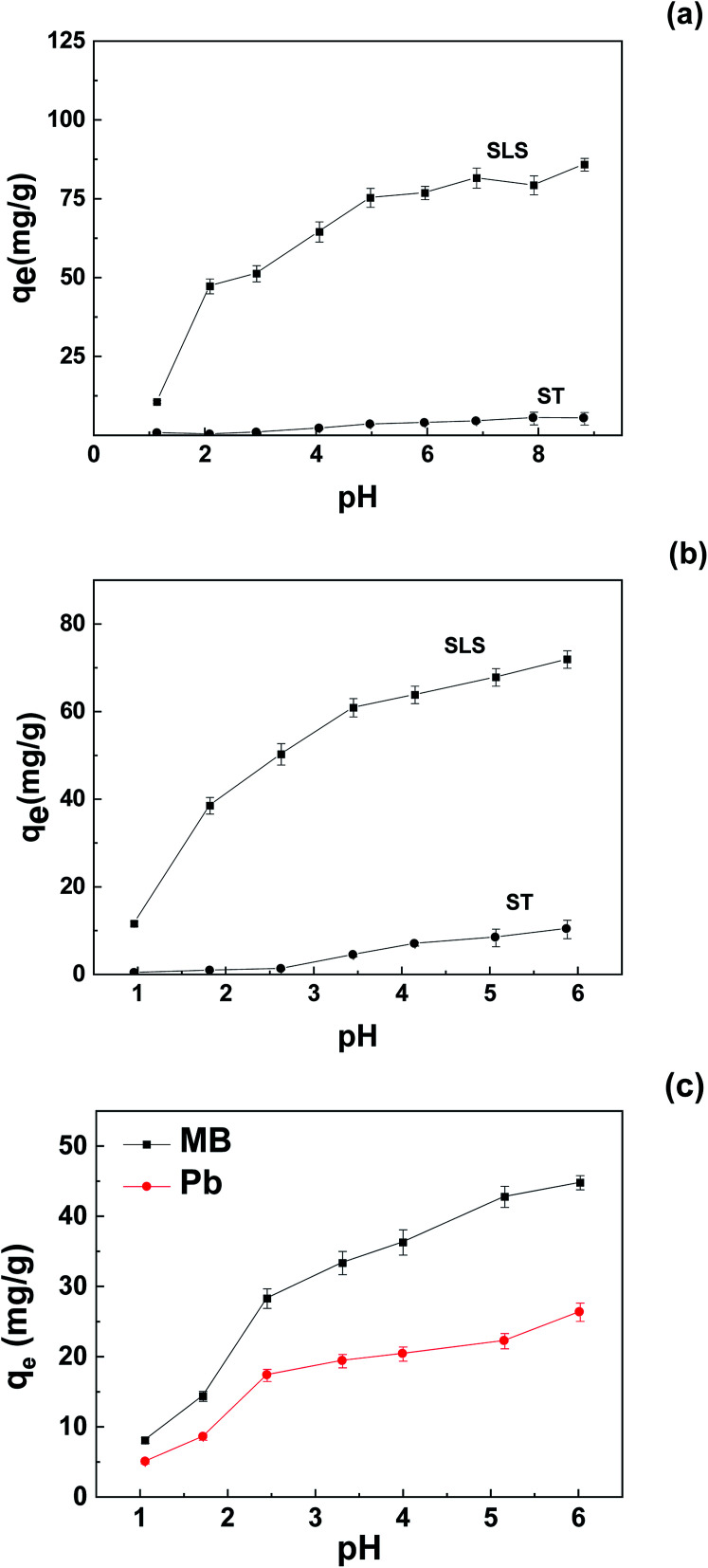
Effect of pH on adsorption of pollutants on SLS and ST: (a) MB; (b) Pb(ii); (c) binary system (initial concentration 400 mg L^−1^, adsorbent 0.05 g).

Fig. 5 showed the trend of adsorption capacity with initial pH. From Fig. 5(a and b), the uptake of both Pb(ii) and MB moderately declined in pH 2–4, the decline became maximal in pH <2. The moderate decline is likely due to protonation of carboxyl groups, sulfonate groups was inhibited only in strongly acidic environment. As the plots suggest, MB adsorption was optimal in pH 5–9, with the maximum adsorption value of about 80 mg g^−1^; For Pb(ii) the optimal range was 5–6, amounting to a peak value of about 70 mg g^−1^. SLS still showed adsorption affinity at pH 1 toward MB and Pb(ii). The trend contradicts those of carboxyl adsorbents,^21^ which is inactive in strongly acidic solutions, apparently this can be attributed to the introduction of sulfonate groups.

Also, according to the sulfonate content of SLS (0.35 mmol g^−1^), at optimal uptake, the molar amount of MB and Pb(ii) made up of 70% and 95% of the molar amount of sulfonates, respectively. The identical molar amount of Pb(ii) and the sulfonate groups indicate a 1 : 1 binding behavior. In addition, the sulfonate groups showed stronger affinity to Pb(ii) than those of MB, if considered in molar uptake.

The adsorption capacity of unmodified starch (ST) was also measured, as the uptakes suggest, the integration with LS enhanced the performance of the adsorbent, the optimal MB uptake of SLS was about one order of magnitude greater for Pb(ii), the uptake change is even greater.

A comparison of adsorption capacity for MB and Pb(ii) between SLS and various adsorbents was listed in Table 2.^22–29^ According to Table 2, some adsorbents are synthesized from chemical reagents, which may bring secondary pollution due to the exhausted adsorbents. The adsorbent of SLS is bio-degradable and eco-friendly deriving from natural-polymer-based materials, though its adsorption capacity is not the highest. Compared with other unmodified natural products, the adsorption capacity of SLS is much higher and can be used as an efficient adsorbent for practical use.

**Table 2:** A comparison on adsorption capacities between SLS and other natural-polymer-based adsorbents

Adsorbent	Adsorbate	*Q* (mg g^−1^)	Ref.
GO/CA	MB	181.81	22
Garlic peel	MB	82.64	23
SLS	MB	80	This work
Sugarcane bagasse	MB	9.41	24
Coir pith carbon	MB	5.87	25
Magnetic chitosan nanopowder	Pb(ii)	113.38	26
SLS	Pb(ii)	70	This work
Pineapple leaves (PL-IDA)	Pb(ii)	44.98	27
Paper industry waste	Pb(ii)	42.4	28
Pineapple leaves (PL-NaOH)	Pb(ii)	18.66	27
Polyaniline grafted chitosan	Pb(ii)	13.23	29

To further evaluate the application potential of the adsorbent in the presence of multiple contaminants, adsorption in binary system was carried out. The results were shown in Fig. 5(c). As demonstrated, competition clearly occurred in solutions where most tests conducted at a pH of 5–6, as the adsorption capacity of MB dropped about 45% and those of Pb(ii) declined more than 60%. According to the trend, the competition in neutral was likely to take place on anionic groups, and MB is more favorable in competition.

#### 3.2.2 Adsorption isotherm study

**Fig. 6:**
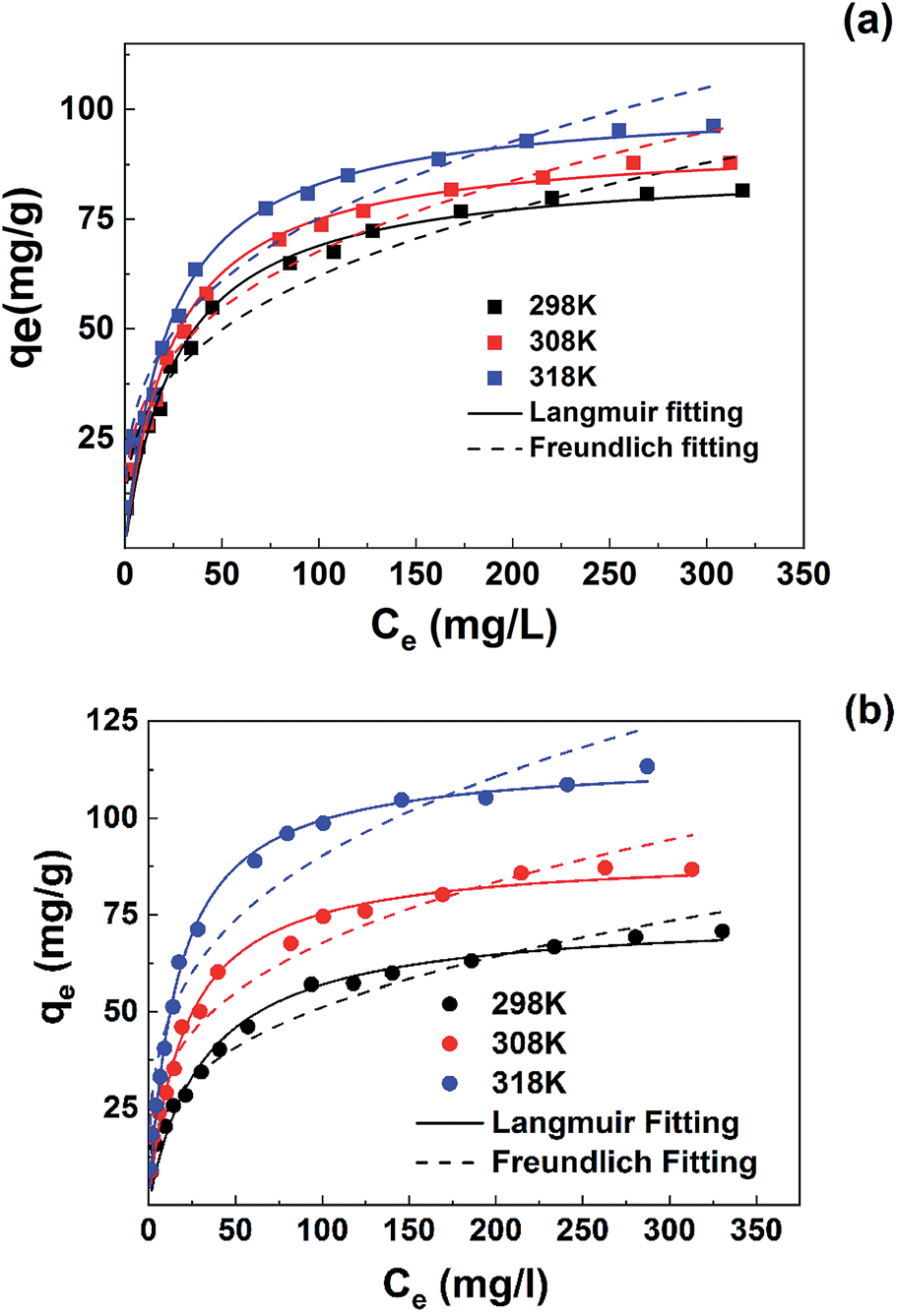
Isothermal equilibrium adsorption of pollutants on SLS at 298, 308 and 318 K with pH 5.0: (a) MB; (b) Pb(ii).

The isothermal adsorption plots of MB/Pb(ii) at three temperatures were shown in Fig. 6.

The adsorption capacity of Pb(ii) and MB both increased along with the rise of temperature, reflecting more favorable adsorption at higher temperature. Also, at high temperatures Pb(ii) uptake increased more than those of MB, at 298 K the maximal uptake of Pb(ii) is still lower than MB, while at 318 K the maximal uptake of Pb(ii) had surpassed that of MB, which indicated a temperature-sensitive preference on the two pollutants.

To further analyze the adsorption behavior and mechanism, Langmuir^30^ and Freundlich^31^ isothermal models were used to fit the data. Non-linear fitting was used for the two models, which can avoid the distortion of data consistency. The models can be represented as follows:4
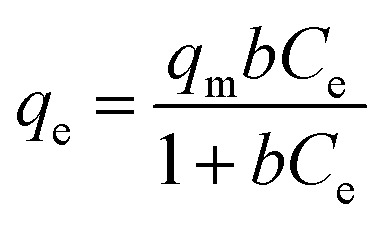
where *q*_m_ (mg g^−1^) is the maximum adsorption capacity when the surface is completely covered by the adsorbates, and *b* (L mg^−1^) represents the Langmuir adsorption constant.

5 *q*_e_ = *k*_F_*C*_e_^1/*n*^

where *k*_F_ and *n* are noted as the Freundlich isotherm constant related to the adsorption capacity and heterogeneity factor, respectively.

The simulation results were shown in Table 3. According to the results, Langmuir model is more agreed with the isothermal adsorption behavior of SLS, indicating a monolayer adsorption.

**Table 3:** Isothermal parameters for the adsorption of MB and Pb(ii) onto SLS

Pollutants	*T* (K)	*q*_m,exp_ (mg g^−1^)	Langmuir model			Freundlich model		
			*q*_m_ (mg g^−1^)	*b* × 10^2^ (L mg^−1^)	*R* ^2^	*K*_f_	*n*	*R* ^2^
MB	298	81.52	87.60	3.70	0.981	14.40	3.15	0.962
	308	87.86	93.30	4.09	0.974	16.41	3.25	0.964
	318	96.28	102.28	4.32	0.968	18.56	3.29	0.955
Pb(ii)	298	70.72	75.00	3.15	0.979	11.31	3.05	0.971
	308	86.72	90.84	4.83	0.984	16.88	3.31	0.954
	318	113.33	115.76	6.12	0.992	23.27	3.40	0.939

Thermodynamic parameters were further derived from *b* of Langmuir model.

6 Δ*G* = −*RT* ln(*Mb*)

7 Δ*G* = Δ*H* − *T*Δ*S*

where Δ*G*, Δ*H* and Δ*S* refer the free energy, enthalpy change and entropy change of the adsorption process, respectively. *R* is the constant of 8.314 J K^−1^ mol^−1^, *M* is the molecular weight of the adsorbate (mg mol^−1^).

According to the simulation results, the value of Δ*G*, being all negative at three temperature, decreases from −23 to −25 kJ mol^−1^ for MB and from −21 to −25 kJ mol^−1^ for Pb(ii). It indicates that the adsorptions are all spontaneous. Δ*H* was 6.09 kJ mol^−1^ for MB and 26.09 kJ mol^−1^ for Pb(ii), respectively. And Δ*S* was 98.5 J mol^−1^ K^−1^ and 160.9 J mol^−1^ K^−1^ for MB and Pb(ii), respectively. The positive value of Δ*H* means that the adsorption processes of MB and Pb(ii) are endothermal, while the positive value of Δ*S* indicate an increase in randomness at the interface of solid/solution during the adsorption processes of adsorbates onto the adsorbents.^32–34^ The Δ*H* and Δ*S* values indicate that the adsorption processes of both pollutants are endothermal ones driven by entropy increase. The higher Δ*H* and Δ*S* of Pb(ii) adsorption also suggest that removal of Pb(ii) is more affected by environmental temperature.

#### 3.2.3 Adsorption kinetics analysis

**Fig. 7:**
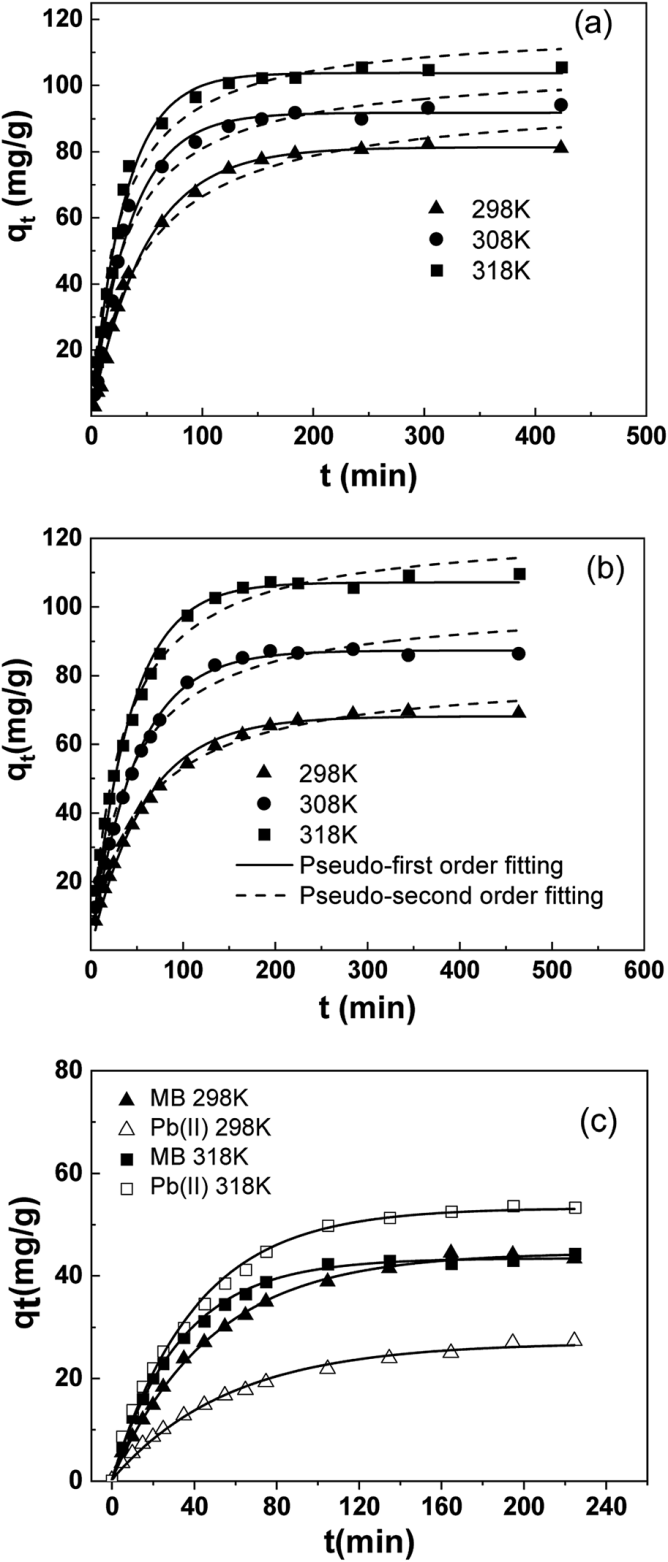
Adsorption kinetics of pollutants on SLS: (a) MB; (b) Pb(ii); (c) binary system.

Data from adsorption kinetics study was shown in Fig. 7. As the data suggest, at 298 K, adsorption equilibrium was reached in about 200 min for both MB and Pb(ii). As temperature rose, the reaction rate increased evidently. At 318 K, equilibria of both adsorbate was reached at about 120 min.

Kinetic results were analyzed using two typical models: pseudo-first^35^ (eqn (8)) and pseudo-second^36^ (eqn (9)) model. To avoid the distortion of data consistency in linear transformation, non-linear fitting for the models was also used. The equations of the models were as follows:

8 *q*_*t*_ = *q*_m_(1 − e^−*k*_1_*t*^)

9
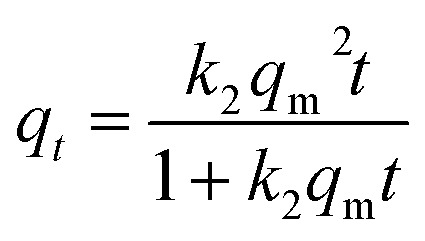
where *k*_1_ (min^−1^) and *k*_2_ (g mg^−1^ min^−1^) represent the adsorption rate constants of pseudo-first-order and pseudo-second-order models, respectively.

According to results, kinetic behavior is in better agreement with pseudo-first order model overall, based on *R*_2_ values. But the value of *R*_2_ in pseudo-second order model is also high, especially for Pb(ii), indicating involvement of multiple mechanisms, which had also been suggested by previous results in Section 3.3.2. Besides, despite relatively fine fitting consistency, the theoretical equilibrium adsorption capacity (*q*_m_) and *k*_2_ from pseudo-second order model did not conform well with the experimental results.

According to Arrhenius Equation, *E*_a_ was calculated and the rate constant was adopted from pseudo-first order model, as those from pseudo-second order model deviates more from experimental data (Table 4).

**Table 4:** Kinetic parameters for MB and Pb(ii) adsorption onto SLS

Pollutants	*T* (K)	*q*_m,exp_ (mg g^−1^)	Pseudo first-order model			Pseudo second-order model		
			*q*_m,cal_ (mg g^−1^)	*k*_1_ × 10^2^ (min^−1^)	*R* ^2^	*q*_m,cal_ (mg g^−1^)	*k*_2_ × 10^4^ (g mg^−1^ min^−1^)	*R* ^2^
MB	298	80.95	81.21	2.03	0.994	96.81	2.23	0.983
	308	88.54	86.28	2.85	0.990	99.52	3.24	0.977
	318	95.10	95.15	3.31	0.993	107.82	3.66	0.978
Pb(ii)	298	68.91	67.97	1.69	0.993	80.77	2.34	0.996
	308	86.35	87.23	2.06	0.995	101.34	2.40	0.984
	318	109.55	107.06	2.37	0.990	122.56	2.40	0.991

According to the fitting results, *E*_a_ of MB was 19.02 kJ mol^−1^ and that of Pb(ii) was 13.30 kJ mol^−1^. The *E*_a_ of both adsorbents is not high (<40 kJ mol^−1^), suggesting a milder temperature dependency of reaction rate compared with most chemical reactions.

To further investigate the adsorption behavior of SLS in complicated systems, kinetics in binary systems was carried out at different temperatures. The results were shown in Fig. 7(c).

According to Fig. 7(c), the adsorption of SLS in binary system showed a clear temperature-sensitive preference, which is in agreement with previous results. At 298 K, removal of MB is preferred over Pb(ii), whereas at 318 K, the preference was inversed as Pb(ii) adsorption became predominant. Due to the inversion of adsorption preference, the equilibrium uptake of MB stay stagnant in binary system at 318 K, still the time for adsorption equilibrium had been shortened considerably.

#### 3.2.4 Discussion on mechanism

**Fig. 8:**
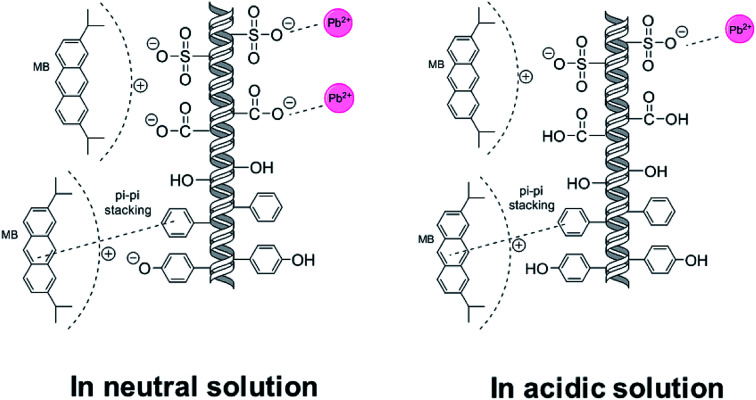
The coadsorption mechanisms in neutral and acidic solutions.

The coadsorption mechanisms in neutral and acidic solutions are illustrated in Fig. 8. The adsorption of MB and Pb(ii) were both achieved through more than one route. Pb(ii) mainly interact with sulfonate and hydroxyl groups on SLS, while MB can be bound on both anionic and aromatic groups due to its aromatic nature.

In addition, according to the isotherm results, adsorption of MB and Pb(ii) on SLS were spontaneous and showed temperature-dependent preference. Adsorption capacity of both pollutants were evidently affected by temperature, while the temperature-sensitivity of Pb(ii) adsorption is more pronounced. Thus the preference of SLS in binary systems was also temperature-dependent, which means the selectivity is tunable by changing temperature.

**Fig. 9:**
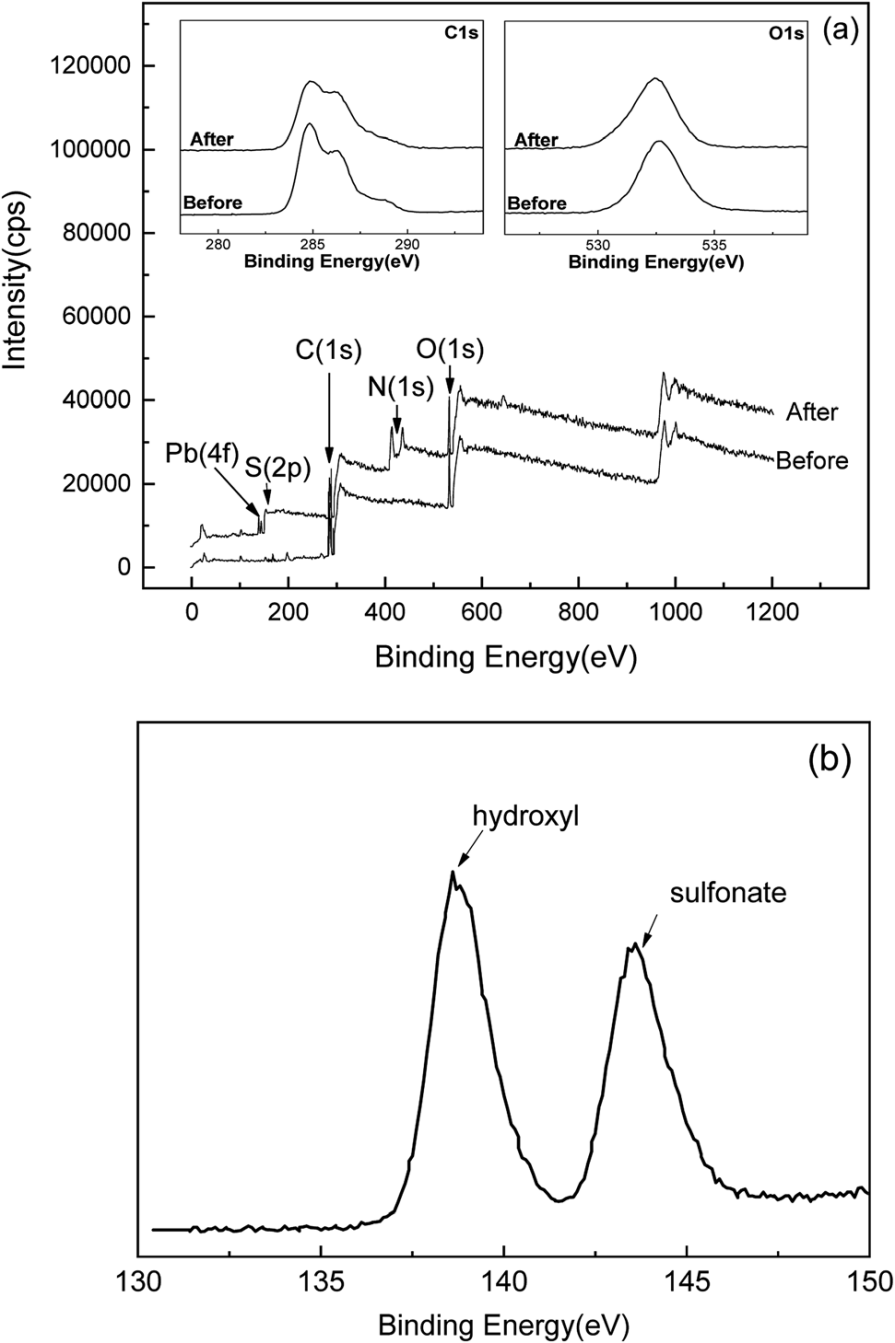
The O 1s and C 1s spectra before and after adsorption and the Global XPS spectra (a); Pb(ii) spectrum after adsorption (b).

The reason for the temperature-dependent behavior lies in microscopic mechanisms. Compared with the molar amount of sulfonate (0.35 mmol g^−1^) on SLS, molar adsorption capacity of Pb(ii) (60–80 mg g^−1^ depending on temperatures) is evidently greater. Therefore, other mechanism beside ion exchange on sulfonate groups must be involved, which may be coordination by hydroxyl groups on ST. Fig. 9(c) showed 4f XPS spectra of Pb(ii) after adsorption, where two peaks can be clearly recognized. The peak at 139 eV is likely from binding with hydroxyl group on ST and the one at 143 eV can be attributed to sulfonate groups,^37,38^ indicating adsorption on two types of groups. Coordination by hydroxyl groups allowed for further uptake increase of Pb(ii) at high temperatures. As for MB, adsorption is also achieved more than a single route, with π–π interaction with aromatic groups on lignin as the other route. As temperature rose π–π stacking is less promoted than coordination by hydroxyl groups.^39^ Therefore the adsorption of MB ends up in less favorability at high temperatures.

### 3.3 Desorption and reusability study

**Fig. 10:**
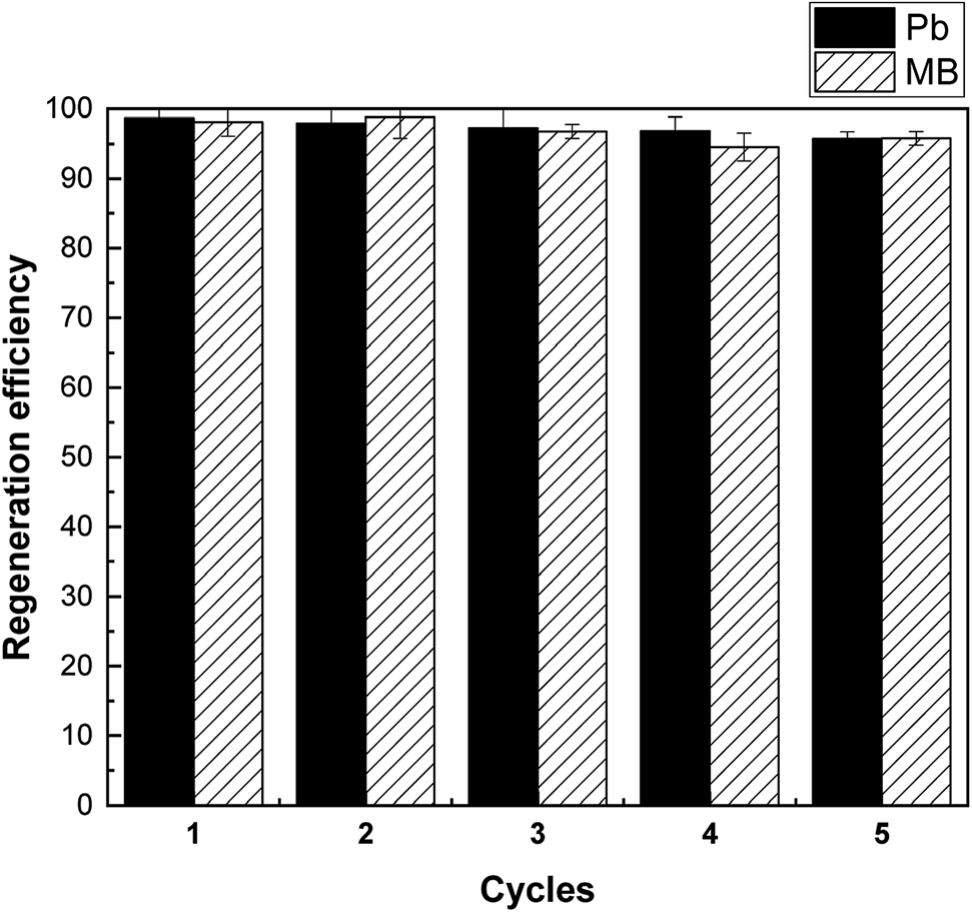
Recovery efficiency of MB and Pb(ii) on SLS.

Regeneration and reuse are another important factors concerning the application potential of adsorbents. From Fig. 10, the adsorption capacity of Pb(ii) and MB only presented slight drop (<5%) in five continuous cycles. The result indicates the satisfactory regeneration of SLS for practical applications.

## 4. Conclusion

In this study, SLS, a fully bio-degradable adsorbent based on lignosulfonate materials, was prepared by suspension-crosslinking method. The application potential for adsorptive heavy metal and dye removal of SLS was assessed using Pb(ii) and MB as model contaminants.

According to the results, SLS was capable of effective adsorption to Pb(ii) and MB. Pb(ii) mainly interact with sulfonate and hydroxyl groups on SLS while MB can be bound on both anionic and aromatic groups due to its aromatic nature. The adsorption behaviors were in agreement with Langmuir and pseudo-first order models and showed temperature-dependent preferences. At 298 K MB was more favorable while at 318 K Pb(ii) adsorption was preferred, indicating a temperature-tunable selectivity. From mechanism aspect, the adsorption of SLS to MB and Pb(ii) were both achieved through more than one route. Recycling and reused experiments showed that used SLS can be readily reactivated and be stably reused. In summary SLS showed great application potential as a bio-degradable and multi-functional adsorbent for heavy metal and aromatic dye removal.

## Conflicts of interest

There are no conflicts to declare.

## Supplementary Material
